# New insights into vitamin D regulation: is there a role for alkaline phosphatase?

**DOI:** 10.1007/s40618-021-01503-w

**Published:** 2021-01-25

**Authors:** G. Bellastella, L. Scappaticcio, M. Longo, R. Carotenuto, C. Carbone, P. Caruso, A. Maio, V. A. Paglionico, M. T. Vietri, M. I. Maiorino, K. Esposito

**Affiliations:** 1 Unit of Endocrinology and Metabolic Diseases, Department of Advanced Medical and Surgical Sciences, University of Campania “Luigi Vanvitelli”, Piazza L. Miraglia 2, 80138 Naples, Italy; 2 Unit of Clinical and Molecular Pathology, Department of Precision Medicine, University of Campania “Luigi Vanvitelli”, Piazza Miraglia 2, 80138 Naples, Italy; 3 Diabetes Unit, Department of Advanced Medical and Surgical Sciences, University of Campania “Luigi Vanvitelli”, Piazza Miraglia 2, 80138 Naples, Italy

**Keywords:** Vitamin D, Alkaline phosphatase, 25-Hydroxyvitamin D, 25-Hydroxylase, CYP2R1

## Abstract

**Purpose:**

The diagnosis of vitamin D deficiency is based on the determination of total plasma 25-hydroxyvitamin D (25-OHD) concentrations, but the regulation of vitamin D 25-hydroxylation is not a major consideration and very little information is available on this activity.

To check what factors could interfere with the activity of vitamin D-25-hydroxylase and thus alter the 25-OHD concentrations, we looked for potential correlations between 25-OHD and results of liver function tests in healthy adults.

**Methods:**

This single-centre study was retrospective and consisted of evaluating the correlations between 25-OHD and the activities of aspartate aminotransferase (AST), alanine aminotransferase (ALT), gamma-glutamyl transpeptidase (GGT), alkaline phosphatase (ALP), and bone alkaline phosphatase (BALP) in 349 healthy subjects aged from 18 to 65 years. In particular, in Group 1 (*n* = 119), we looked for correlations between 25OHD and all liver function tests and in Group 2 (*n* = 230) the correlation between 25OHD and BALP.

**Results:**

In Group 1, we found no correlation between 25OHD and AST (*r* =  − 0.03; *p* = 0.8), ALT (*r* =  − 0.02; *p* = 0.91), GGT (r =  − 0.08; p = 0.68), direct bilirubin (*r* =  − 0.02; *p* = 0.89), indirect bilirubin (*r* =  − 0.24; *p* = 0.21), and total bilirubin (*r* =  − 0.24; *p* = 0.21) but one between 25OHD and ALP (*r* =  − 0.2; *p* = 0.007); in Group 2, we found a significant negative correlation between 25-OHD and BALP (*r* =  − 0.2; *p* = 0.0008).

**Conclusions:**

The correlations that we found suggest that ALP and BALP might be involved in the regulation of vitamin D-25-hydroxylase activity, but further studies are mandatory to confirm our assumptions.

## Introduction

Vitamin D, historically associated with the regulation of calcium metabolism at the bone level, has been recently demonstrated to be strongly involved in many biological processes [1–9]. Vitamin D exerts its action at cellular levels by interacting with the nuclear vitamin D receptor (VDR), belonging to the superfamily of nuclear receptors modulating gene transcription.

Vitamin D is unique among hormones, because it can be made in the skin from exposure to sunlight. It comes in two forms: vitamin D2 (ergocalciferol) and vitamin D3 (cholecalciferol). The former was the first to be isolated and is obtained from the UV irradiation of the plant sterol ergosterol. The latter is produced in the skin from 7-dehydrocholesterol by ultraviolet (UV) radiation and is present in oil-rich fish such as salmon, mackerel, and herring. Vitamin D that comes from the skin or diet is biologically inert and requires first hydroxylation by vitamin D-25-hydroxylase (25-OHase, CYP2R1) to 25-hydroxyvitamin D (25-OHD), the main circulating form [6, 9]. In human adult and fetal tissues, CYP2R1 mRNA is ubiquitous; 25-OHase expression has been observed in dermal fibroblast and in prostate cancer LNCaP cells, but its expression is primarily in the liver and testes [10–12]. Our previous results of lower vitamin D concentrations and higher prevalence of Vitamin D deficiency in diabetic patients with hypogonadism compared with patients without hypogonadism supported the role of 25-OHase in the testis [13].

25-OHD is then further metabolized in the kidneys to 1,25-dihydroxyvitamin D [1,25(OH)2D] by the enzyme CYP27B1. 1,25(OH)2D is the ligand for the vitamin D receptor (VDR), a transcription factor modulating the activity of many genes involved in the regulation of calcium, phosphate, and bone metabolism.

Vitamin D deficiency and insufficiency is a global health issue that afflicts more than 1 billion children and adults worldwide [14], and seems to be associated with increased prevalence of some diseases including neuropathy, malignancy, infertility, cardiovascular diseases, kidney diseases, glucose metabolism, and immunological dysfunctions [1]. There is no agreement yet on “normal levels” of circulating 25-OHD, but there is agreement to treat all subjects with serum 25-OHD levels < 20 ng/mL (50 nmol/L) with vitamin D [15]. The estimated prevalence of vitamin D deficiency in adult population depends on its cut-off definition [16, 17]. The National Health and Nutrition Examination Survey (NHANES, 2001–2006) showed that 25% of the population was at risk of insufficiency, as defined by serum 25-OHD levels of 12–20 ng/mL, and that 8% had very low 25-OHD levels (< 12 ng/mL) [18].

Since the diagnosis of vitamin D deficiency is based on the determination of total plasma 25-OHD concentrations, but the regulation of vitamin D 25-hydroxylation is not a major consideration, and very little information is available on this activity, we planned this study to investigate if some factors interfere with the activity of CYP2R1 and with 25-OHD concentrations. In particular, we focused our attention on the potential correlations between 25-OHD and results of liver function tests in healthy adults.

## Patients and methods

The retrospective study was carried out at the University Hospital “L. Vanvitelli”, Naples, Italy. We searched in the database of Clinical and Molecular Pathology Unit for all healthy controls of previous studies [4, 13] who were also screened for vitamin D status and liver function (Group 1, *n* = 119); we also recruited other healthy people (University and Hospital staff and donors) who underwent the screening for vitamin D and bone alkaline phosphatase between January 2015 and December 2019 (Group 2, *n* = 230). On the basis of these criteria, we enrolled 349 subjects, including 157 males and 192 females. All people had to be aged from 18 to 65 years. Obesity, consumption of fish oil, vitamin D, mineral supplements or any drug interfering with vitamin D metabolism were considered exclusion criteria. The study was acknowledged by the Ethics Committee of the University of Campania and AORN Ospedali dei Colli.

Assays for aspartate aminotransferase (AST), alanine aminotransferase (ALT), gamma-glutamyl transpeptidase (GGT), alkaline phosphatase (ALP), bone alkaline phosphatase (BALP), direct bilirubin, indirect bilirubin, total bilirubin, and 25-OHD were performed in the hospital’s chemistry laboratory.

25-OHD levels were measured with a chemiluminescence method (LIAISON®, DiaSorin, Stillwater, USA). The LIAISON 25OHD total assay measures concentrations between 4.0 and 150 ng/ml with 7.8% intra-assay coefficient of variation. Although deficiency and insufficiency cut-off points are still a topic of discussion, in this study, vitamin D deficiency was diagnosed when serum 25-OHD was < 20 ng/ml. We measured the circulating serum 25-OHD level, which represents the major circulating vitamin D metabolite and is a reliable indicator of vitamin D status. Because of interference by seasonal variations, we enrolled subjects who underwent vitamin D screening in each season, to assess the correlations along all the year.

ALP and BALP assays were, respectively, assessed by Alkaline Phosphatese 7D55 Architect system (Abbott, Wiesbaden, Germany) and by LIAISON® BAP OSTASE® (DiaSorin, Stillwater, USA).

In the first group of 119 (69 F, 50 M) subjects, we looked for correlations between 25OHD and all liver test enzymes. Subsequently, we analysed the second group of 230 (123 F, 107 M) subjects in which we evaluated only the correlation between 25-OHD and BALP.

Results are presented as mean ± standard deviation, median, and interquartile range or number and percentage. Correlations between 25-OHD levels and liver tests or BALP were evaluated by Pearson or Spearman Test. A value of *p* < 0.05 was considered statistically significant. All statistical analysis were performed using SPSS software (version 10.05, SPSS, Chicago, IL, USA).

## Results

**Fig. 1:**
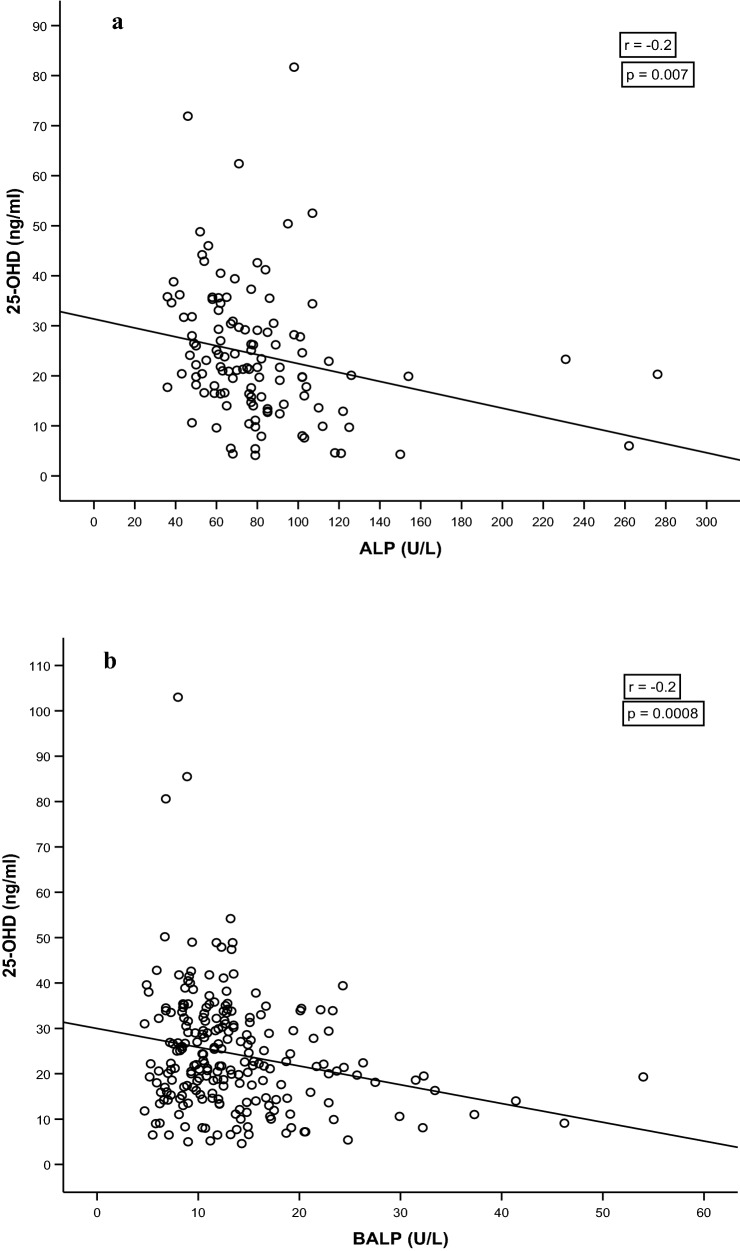
Correlation between 25-OHD and ALP (**a**) and 25-OHD and BALP (**b**)

The demographic and biochemical characteristics of the study population are reported in Table 1. The mean age was 46.7 ± 20.4 years. In the overall population, we found a mean 25OHD level of 24.4 ± 13.1 ng/ml. 146 out of 349 (41.8%) subjects had vitamin D deficiency with 60 (17%) subjects with very low 25OHD concentrations (< 12 ng/ml). 25OHD levels were significantly higher in the summer–autumn period (25.8 ng/ml; IR: 18.8–33.5) than in the winter–spring period (20.4 ng/ml; IR: 13.4–30.3; *p* < 0.001). In Group 1 (*n* = 119), we found no correlation between 25OHD and AST (*r* =  − 0.03; *p* = 0.8), ALT (*r* =  − 0.02; *p* = 0.91), GGT (*r* =  − 0.08; *p* = 0.68), direct bilirubin (*r* =  − 0.02; *p* = 0.89), indirect bilirubin (*r* =  − 0.24; *p* = 0.21), and total bilirubin (*r* =  − 0.24; *p* = 0.21) but a correlation between 25OHD and ALP (*r* =  − 0.2; *p* = 0.007) (Table 2a) (Fig. 1a); in Group 2 (n = 230), we found a significant negative correlation between 25-OHD and BALP (*r* =  − 0.2; *p* = 0.0008) (Fig. 1b). In a subgroup analysis of Group 2, we assessed the correlation in people aged > 18 ≤ 40 and in people > 40 ≤ 65, in males and females and on the basis of the season in which the sample was drawn: correlation between 25-OHD and BALP remained significant in all the subgroups (Table 2b).

**Table 1:** Demographic and biochemical characteristics of the study population

	Study population
	*N* = 349
Age (yr)	46.7 ± 20.4
Sex (% of men)	45
25(OH)D (ng/ml)	21.7 (14.6–31.4)
Summer–autumn (ng/ml)	25.8 (18.8–33.5)
Winter–spring (ng/ml)	20.4 (13.4–30.3)
< 20 ng/ml, *n* (%)	146 (41.8%)
< 12 ng/ml, *n* (%)	60 (17%)*

	Group 1
	*N* = 119
25(OH)D (ng/ml)	21.7 (15.8 to − 30)
AST (U/L)	18.0 (15–23)
ALT (U/L)	17.0 (13–27)
GGT (U/L)	20.0 (15–28)
ALP (U/L)	78.5 (61–94)
Total bilirubin (mg/dl)	0.73 (0.50–1.1)
Direct bilirubin (mg/dl)	0.31 (0.25–0.44)
Indirect bilirubin (mg/dl)	0.43 (0.25–0.63)

	Group 2
	*N* = 230
25(OH)D (ng/ml)	22 (15–32)
BALP (U/L)	11.9 (9.0–15.5)

**Table 2:** (a) Correlations between 25-OHD and liver function test results in Group 1. (b) Correlation between 25-OHD and BALP in subgroups of Group 2 (for seasons, sex and age)

(a)														
Group 1	AST		ALT		GGT		ALP		Total bilirubin		Direct bilirubin		Indirect bilirubin	
	*r*	*p*	*r*	*P*	*r*	*p*	*r*	*p*	*r*	*p*	*r*	*p*	*r*	*p*
25-OHD	− 0.03	0.8	− 0.02	0.91	0.08	0.68	− **0.2**	**0.007**	− 0.24	0.21	− 0.02	0.89	− 0.24	0.21

(b)												
Group 2	Summer–Autumn		Winter–Spring		Males		Females		≤ 40		> 40	
	*r*	*p*	*r*	*p*	*r*	*p*	*r*	*p*	*r*	*p*	*R*	*p*
25-OHD–BALP	** − 0.2**	**0.004**	** − 0.2**	**0.002**	** − 0.2**	**0.04**	** − 0.2**	**0.0003**	** − 0.3**	**0.03**	** − 0.27**	**0.02**

The significant correlations are in bold

*r* correlation coefficient

We also observed a seasonal variation of both ALP and BALP concentrations with higher levels in the summer–autumn (ALP 79 U/L; IR 67.2–92; BALP 12.4 U/L; IR 9.5–16.2) than in winter–spring (ALP 77 U/L; IR 61.2–92.5, *p* = 0.81; BALP 12.4U/L; IR: 9.5–16.2, *p* = 0.04).

## Discussion

To the best of our knowledge, this is the first study evaluating the correlations between 25OHD and liver function tests in healthy subjects. We found a significant negative correlation between 25OHD concentrations, ALP and BALP but not with other liver enzymes.

Alkaline phosphatase (ALP) is an enzyme found in several tissues throughout the body. It can originate from the liver, bone, intestines, or kidneys, but bone ALP and liver ALP constitute about 95% of the total ALP activity in human serum. Elevated levels of ALP in the blood are most commonly caused by liver disease or bone disorders. Bone alkaline phosphatase (BALP) is an ectoenzyme attached to the outer surface of the cell membrane of osteoblasts by glycosylphosphatidylinositol. It is a major regulator of bone mineralisation, hydrolysing inorganic pyrophosphate, a natural inhibitor of mineralisation, and providing inorganic phosphate for the synthesis of hydroxyapatite. It is partially released into the circulation. The BALP and ALP levels will rise in the serum following increased production by osteoblasts in a state of high bone turnover. Throughout life, bone continuously remodels itself through bone resorption and replacement. Remodelling results from the action of osteoblasts and osteoclasts and each remodelling cycle consists of three consecutive phases: resorption, reversal, and formation. The resorption phase is characterised by the migration of partially differentiated mononuclear pre-osteoclasts to the bone surface where they form multinucleated osteoclasts. After the completion of osteoclastic resorption, there is a reversal phase when mononuclear cells appear on the bone surface. Then, new osteoblasts begin bone formation and BALP is considered a good marker of this phase. Resorption probably continues for about 2 weeks, the reversal phase may last up to 4 or 5 weeks, while formation can continue for four months until the new bone structural unit is completely created [19].

On the basis of our results and the physiology of bone remodelling, we hypothesize a complex mechanism in which in the resorption phase, low osteoblast activity and low release of BALP stimulate 25-OHase activity; on the contrary, higher BALP levels of the formation phase inhibit the activity of CYP2R1. Although previous studies showed ALP concentrations significantly higher in individuals with vitamin D deficiency [20] and a negative association between BALP and 25-OHD in 58 patients (median age: 62 years) with gastric cancer [21], no previous study has considered this issue in healthy subjects.

This study has the benefits of a large healthy population in a wide range of ages to assess the correlation between 25-OHD and ALP and BALP. Moreover, the opportunity of considering samples drawn during all the year allowed to assess better the persistence of the correlation in different seasons. Indeed, our study confirms both the well known seasonal oscillations of vitamin D concentrations with significantly higher levels in the summer–autumn period than in winter–spring and the persistence of correlation between 25-OHD and BALP in different seasons. Moreover, we found that BALP also showed a seasonal variability, as does vitamin D, with higher levels in summer-autumn.

On the basis of our results, we speculate that assessing the real vitamin D status of subjects is important, considering not only the seasonality of vitamin D but also other mechanisms of bone remodelling.

This study has certain limitations, including that all data were examined retrospectively and the correlation with ALP and BALP was assessed in two different groups. Moreover, we assessed only correlations, but we have not experiments showing that one factor interferes with another.

Further prospective longitudinal studies aimed at evaluating the vitamin D status of each subject from samples drawn at different times of the year and experiments to verify a causal relationship between 25-OHD and ALP/BALP are needed to confirm our assumptions and to study the opportunity of a cyclic replacement therapy in the case of vitamin D deficiency.
